# Students’ and educators’ perceptions of clinical academic pathways in the UK: a qualitative study

**DOI:** 10.1136/bmjopen-2024-089791

**Published:** 2025-03-24

**Authors:** Cinzia Greco, Chiu-Yi Lin, Natalie J Gardiner, Gabrielle M Finn, Adam Danquah, Hema Radhakrishnan

**Affiliations:** 1 Faculty of Biology, Medicine and Health, The University of Manchester, Manchester, UK

**Keywords:** MEDICAL EDUCATION & TRAINING, QUALITATIVE RESEARCH, Health Workforce

## Abstract

**Abstract:**

**Objectives:**

This study aimed to understand the experiences and perceptions of medical students and medical educators regarding barriers and facilitators for pursuing clinical academic pathways.

**Design:**

A qualitative study using semistructured interviews and focus groups was conducted. A reflexive thematic analysis was used for data analysis. Theoretical and snowball sampling approaches were applied for participant recruitment.

**Setting:**

University of Manchester and NHS Trusts in the Greater Manchester region.

**Participants:**

The sample of this study was composed of 43 participants, including medical and dental students, clinical academics, clinicians and stakeholders.

**Results:**

Three themes were identified: Theme 1: Knowledge of the CA pathway; Theme 2: The costs linked to pursuing a clinical academic pathway; Theme 3: Exposure to and time for research.

**Conclusions:**

While institutions offer opportunities for research experience, there is a need for extended and more tailored opportunities and information, and the overall cost of training for students reduces the attractiveness of the clinical academic pathway.

**STRENGTHS AND LIMITATIONS OF THIS STUDY**

- We are able to compare students’ perspectives with those of clinical academics, clinicians and stakeholders, enriching the overall findings.
- We employed a novel qualitative interpretative frame to analyse the data, which combines thematic analysis and extended case method.
- Participants were limited to medical and dental students, doctors and dentists, and the results might not be generalisable to other medical professions.
- The study is limited to a single university (for what concerns the student participants) and to the Greater Manchester area (for the other participants), and the generalisability to other contexts should be tested.

## Introduction

In the UK, a clinical academic (CA) is defined as a clinician who is professionally trained and practising, and works as a researcher or lecturer, generally employed in a university or research institution. There are well-established clinical medical academic pathways (https://www.catch.ac.uk/example-medical-clinical-academic-training-pathway) in many countries, including the UK^1^ and Australia.^2^ The UK Clinical Research Collaboration (UKCRC)^3^ has set objectives and developed principles for CA careers, including coordinating of training opportunities, establishing contract structures that allow combining research and the clinic, mentoring schemes and increasing funding.

It is widely accepted that CAs are central to innovation in health research and ensuring quality of care.^4^ CAs provide expertise in patient-based research, likely to have an impact in the real world.^5^ They play a key role in the implementation of research results into clinical practice. CA training provides a unique opportunity which enables healthcare professionals to simultaneously develop their clinical and research skills.^3 6^ To build long-term trustful relationships with disadvantaged communities and reduce health inequalities through innovations in healthcare research, it is important that our CA cohort represents the diversity of the population it serves.^7 8^ It is evident that women and individuals from ethnic minority backgrounds are still under-represented in the CA pathways.^9^,^13^ This means that access to CA careers needs to be improved in order to have not only a sufficient level of staffing but also a diverse group of CAs that can best serve the population.

CA pathways require dedicated planning and opportunities for career progression from undergraduate to postdoctoral levels.^14^ Those in medicine and dentistry share similar routes and frameworks for CA training schemes,^4^ Health Education England 2015, including the National Institute for Health and Care Research (NIHR) Academic Clinical Fellowships and NIHR Clinical Lectureships in the UK.^4^

The centrality and the importance of CAs have been recognised.^15^,^17^ While recent interventions have made it possible to increase the number of CAs, difficulties in recruitment and retention remain^1618^,^20^ and need to be further explored. Different pathways can be followed to pursue a CA career; some are more structured, while others are more opportunistic and informal and can depend on opportunities and experiences encountered by professionals in their careers.^21^,^23^ The role of medical education in improving the recruitment of CAs and in widening the opportunities to choose this pathway is central. While there are opportunities to enter the CA pathway later in one’s career, ensuring medical students an early and systematic understanding of clinical academia and research can improve recruitment and offer more equal opportunities.^24 25^ Medical education and mentorship from lecturers are the main sources of information about the possibility of adding a research component to the medical career, and opportunities to start building a research profile are already present in the first years of a medicine course. While there is a particular emphasis on CAs as researchers, CAs have an equally important role in teaching.^16^ Much of what has been written about the advantages and challenges of having a research career with a clinical one also applies to combining a clinical career with a teaching one. Clinical lecturers are valuable mentors, educators and role models and are critical to informing and facilitating students to pursue a CA career.

In a previous analysis conducted on the same set of data presented here, barriers and facilitators were identified for those already in different stages of the CA pathway^26^ and which highlighted the importance of visible CA role models, mentoring and financial support for students.^26^ This current study aims to understand the experiences and perceptions of medical students and medical educators regarding perceived barriers and enablers for pursuing CA careers. In particular, as early exposure to research and academic opportunities emerged as a key factor in the first analysis, we examine here to what degree medical education can help improve access to CA pathways.

## Methods

### Design

A qualitative study using semistructured interviews and focus groups to understand the experiences of medical and dental students and educators (CAs, clinicians, stakeholders) was conducted in Greater Manchester. The aim of this study was to explore their perspectives on the CA pathway, including the role of medical education and training, as previously described.^26^ The research questions of the general project focused on the experiences and barriers of the CA pathway, particularly for minoritised profiles. In this article, we focus on the experiences and barriers specific to medical education. Given the relatively limited size of the populations of reference and the exploratory nature of the project and research questions, a qualitative approach was deemed to be the best methodological choice.

### Participants and setting

Theoretical and snowball approaches^27^ to sample and recruit participants working in Greater Manchester were used. Stakeholders involved in the management of CA careers, and medical and dental students enrolled in years 3–5 at the University of Manchester were contacted. CA training networks advertised the study, the academic networks of the research team were approached, and social media and internal mailing through the medical school were used to advertise the study.

### Data collection

Participants were provided with an online participant information sheet, and those who chose to participate gave both written consent before the interview and oral consent at the start of the interview. We conducted individual semistructured interviews with the CAs, clinicians and stakeholders, and two focus groups were conducted with medical and dental students. Both the interviews and the focus groups were held online through Zoom or Microsoft Teams. A general interview/focus group guide was provided according to the profile of the participants. These included questions about the participants’ experience of (or, in the students’ case, knowledge about) the CA pathway, potential barriers and facilitators to enter the pathway and the broader experience of the research and teaching culture in the institutions in which they had spent time. We followed the principle of information power^28^ to determine when the number and diversity of interviewees allowed us to reach a rich understanding of their experiences. The first two authors (CG and C-YL) conducted all interviews and focus groups; the two researchers are experienced qualitative researchers working at the same institution as most of the participants, but not involved in medical training and with no previous relations with the participants, which means that participants were not limited in their expression by dual relations with the interviewees. The remaining authors are all involved in medical education, and one (AD) is a CA, which allowed them to contribute an internal point of view to the analysis.

### Data analysis

Interviews and student focus groups were recorded and transcribed. We analysed the data by applying Braun and Clarke’s^29^ reflexive approach to thematic analysis, which guided the main process of establishing and applying codes and identifying themes. The authors involved in the analysis engaged in reflexive conversations about the meaning of the data and the themes that could provide a theoretical understanding of them. In particular, the content of the interviews has been analysed first independently and then in a process of comparison by the two authors involved in data collection (CG and C-YL) to ensure that the meaning emerging in the interviews was identified and then discussed with the other authors to check for accuracy of the interpretation against internal knowledge of the organisation of medical education in the institution. We further applied Burawoy’s extended case method approach^30^ to connect our data with existing theories about the student and CA experience and with dimensions of the organisation of clinical and academic work in the UK beyond those directly explored in the interviews.

## Patients and public involvement

This research did not involve patients but only medical personnel and students; patients were not involved in the design, or conduct, or reporting, or dissemination plans of this research.

## Results

### Characteristics of participants

Data collection was carried out between September 2022 and February 2023. Overall, 43 participants took part in either an interview (37 CAs, clinician and stakeholder participants) or a focus group (6 medical and dental student participants). 21 men and 22 women with a wide range of roles were interviewed for this study (19 CAs, 5 clinicians, 13 stakeholders and 6 medical or dental students). The student participants were in their 20s, while the other participants were aged between 30 and 70 (9 in 30–39, 7 in 40–49, 18 in 50–59 and 3 in 60+). Of the overall group of 43 participants, 32 were White, 9 Asian and 2 Black.

### Themes

Three themes were generated: Theme 1: Knowledge of the CA pathway; Theme 2: The costs linked to pursuing a CA pathway; Theme 3: Exposure to and time for research.

#### Theme 1: Knowledge of the CA pathway

The students who participated in our research were provided with information on CA careers during their medical/dental education. However, the quality and quantity of information varied: while students were aware of what a CA career pathway was, there was evidence of uncertainty around the ways to pursue a CA career. When asked if they would be interested in a CA career, the lack of more detailed information played a role in shaping their answers:

‘We’ve not had information at all. One thing that most dental students find is that we actually…we’re just very under prepared with all the opportunities that are out there for us after dental school. I think most of the things that we find are resources through societies or voluntary speakers. But with regards to this, I’ve never been told anything.’ (S1)

Q: ‘Would you be interested in pursuing a medical academic career?’

A: ‘I think so, but again, I feel like we’re all just quite in the dark about it. So, I couldn’t say for certain in all honestly, because I feel like I don't know the pros and cons about it or what it actually entails. I definitely don't feel like I could give a definite answer right now.’ (S2)

Students were also aware of how changes in the organisation of their study programmes can have repercussions that are difficult to predict.

‘We get told a bit of information from year one about it when we do a little group project focusing on a specialty, and with the option…with a little description, ‘oh, if you’re further interest in pursuing a specialty this will be beneficial for your future if you want to consider intercalation during your time’. However, with changes to the foundation programme intercalated degrees no longer offer points for the foundation programme. So, I think it’s definitely made me happy that I didn’t pick to do one but I don’t know the impact of this to my future career, especially if I’m considering going down this clinical academia path.’ (S2)

In this extract, the reference is in particular to the fact that intercalated degrees do not contribute anymore to the ranking students can obtain when applying for foundation programmes, reducing their attractiveness.

The importance of the CA pathway was also recognised by the CAs and stakeholders we interviewed. Many underlined that one of the biggest difficulties concerning this career pathway is ensuring that students are informed about the possibility of pursuing this path early on in their education. One of the aspects highlighted was the selective and competitive nature of the CA path and how students could benefit from greater knowledge of the path at the beginning of their studies.

‘Personally I think it should be from the very beginning that it should at least be the information should be available there. And the reason that I think that is because having an academic career and getting yourself established on that pathway, you really have to think about milestones. How you can evidence things because it is quite competitive. Like every stage, there’s always quite a competitive selection process and interview, and the things that they look for is commitment to it.’ (CA10)

‘We probably need to be doing more science and lab-based projects with the students right from the start. […] You know doing that from an early stage and trying to capture them, and really, you know, make them realise what a clinical academic is.’ (CA16)

‘My main concern really is it is about the future. How we get that across to medical students? So you have to create the pipeline to be coming through and one of the biggest things that has changed in the last couple of years is […] for intercalating medical students. Those costs have increased and so that puts students off on the background of already having large student loans […]. I think about how do we expose the medical students to the opportunities to research when the medical curriculum is expanding so much that you have to cover all of that? There’s, you know, research seems like a luxury, and yet we're in danger of not having the pipeline of people coming through in the future.’ (CA18)

The interviews conducted with the students and those conducted with other participants show interesting overlaps, and they both underline how crucial it is to provide information early to students. But they also underline that another important element is the quality of this information. Students need to be exposed to CA and the opportunities to conduct research to process the information about CA pathways.

#### Theme 2: The costs linked to pursuing a CA pathway

Another aspect highlighted in our data is the cost of studies that students have to face and which, in some cases, acted as a deterrent and discouraged students from less privileged backgrounds from undertaking an intercalated degree which could orient medical studies towards a research career. In the focus groups with students, there was considerable discussion about the costs of intercalated degrees. Taking an intercalated degree comes with costs in terms of additional student fees and also delaying employment and earning a salary. With many medical and dental students already needing to take part-time jobs during their studies, such delays might be problematic:

‘It is the financial aspect, I think, having already done a degree.

There’s opportunities afterwards in a paid position instead of paying [for it] yourself.’ *(S5*)

A student further said that they considered research ‘an indulgence’ to delay the start of their work life to conduct a PhD. Financial burdens and their impact were also highlighted by the experiences of early career CAs:

‘I was waitressing, working in a cinema, working in anything and everything, I was working as a receptionist all throughout med school’ (CA04)

The financial difficulties in pursuing a CA career were highlighted by other interviewees as well:

‘[I] think there’s a bit of a cost of living crisis for students so it’s extending…so it’s not just extending your student life by a year and therefore being a student is much more expensive than it used to be so there’s that but then you’re also deferring starting earning for a year so it’s not just the extra money that you pay, it’s the fact that you don’t get your salary for an extra year. So I think there is a big inequality there. It tends to be people with the families that can afford the extra year, so I think there’s quite a big inequality in intercalation.’ (G06)

#### Theme 3: Exposure to and time for research

In addition to being informed about the CA pathway and being able to afford the costs, the students involved in the focus group discussed how having the opportunities to be exposed to research could be a further factor in helping to start a CA career. Some students underlined that they had the opportunity to carry out research, but they also stressed that this possibility was linked to an active search for opportunities on their part.

‘I think it’s I’ve really sought out the people and the opportunities through the degree to make that happen, because […] that was a big aim for me. A big goal going into the degree.’ (S4)

‘I think like personally, I feel been quite lucky and had like opportunities to get involved in research while I’ve been at Manchester. But I kind of wanna say I think that is because I'm a grad[uate student]. I think that is because I've had exposure to research before and I think that was probably quite like self-motivated and or just that I maybe had the confidence to approach the PEP and the APEP with that kind of attitude. And knowing what I wanted to get out of it.’ (S3)

The Personal Excellence Pathway (PEP) and Applied PEP (APEP) mentioned by S3 are programmes specific to the University of Manchester (respectively, in the second and third year of medicine), through which students are exposed to research by completing individual small-scale projects. While such institutional arrangements can be conducive to socialising students to CA careers, there is still concern about whether, in reality, there is sufficient and protected time for research in the CA role.

‘So now I’m in clinical there’s a lot more focus on the clinical aspects of medicine and being with the patient. It’s why I did the course. But there’s also this divide between…perceptions between, I guess, academics vs clinicians that academics are there to churn out papers on the institute. and if they don’t get out a certain number of publications within a time, they might lose their positions at the university, and it is quite pressurising that way. And if say, someone wanted to be a clinician academic, they already have enough clinical pressures, it can be quite off-putting is the impression that I seem to get.’ (S2).

’[I think that] all of us and especially now [are] considering the different stresses on a clinical career and that face you at different points, whether […] the pressure of the health system or something in order to do research. I feel like you kind of need to have a baseline level of wellbeing and like have a good work life balance within your clinical job let alone to extend it to do research. You kind of need to have like extra resources and […] there’s talk about institutions protecting the time for research and institutions not protecting the time for research, and that’s big deterrent.’ (S3).

The potential difficulties in balancing the clinical part of training with the scientific dimension were mentioned by the students involved in the focus groups. This further intersected with the perceived stress posed on the healthcare system and the demands of personal life.

## Discussion

### Main findings

In this study, we wanted to explore the opinions students and educators have of the CA pathways. Across our analysis, we identified three themes discussing resources that help individuals pursue a CA career and perceived obstacles when these resources are in short supply: information regarding the CA pathway, economic resources to fund (additional) training and exposure to research. Our research has shown that students receive information about the CA pathway at the institution where we conducted the study. Additionally, research-focused components of medical training, such as PEP and APEP, can provide individuals with valuable exposure to research. However, the participants have highlighted that not all students are in a position to benefit equally from exposure to research. First, our respondents were clear that they had research opportunities also because they were proactive in looking for them, suggesting that other students would need both more exposure and more opportunities. Furthermore, the direct and indirect cost of undergoing additional training emerged as a critical factor from both the students and educators as a major barrier, given the cost of university fees and the recent rise in the cost of living can be disincentives to pursuing the additional training needed for a CA career. In the UK medical education system, one important opportunity to start a CA career has been the possibility of undertaking an intercalated degree. An intercalated degree means pausing medical or dental study for a year or more to study another subject—through, for example, a BSc, MPhil or a PhD. Students pursuing an intercalated degree usually reported positive experiences.^31 32^ However, recent revisions that have removed educational achievement (including intercalated degrees) from the factors improving the score when applying for foundation posts have reduced the advantages of completing an intercalated degree.^33^

### Strengths and weaknesses of the study

While a limited number of focus groups were undertaken with undergraduate medical and dental student participants, the emphasis of this study was to understand the whole experience of clinical training based on both the students’ and the educators’ perceptions and lived experiences. The data show a rich array of information from early career clinicians and CA as they were able to provide reflective insights into the training and information they received and their career progression in different pathways.

### Our findings in relation to other studies

Other recent studies have explored the barriers and facilitations to access CA careers for medics and dentists, including several studies conducted in the UK^1620 22 23 34^,^37^ and one study conducted in Denmark.^38^ While some studies^1622 34^,^36^ were based on a larger number of respondents or involved multiple institutions, the other were in line with the one presented here in focusing on a single institution/area and in terms of number of participants. Among the studies we identified, none involved medical and dental students, with PhD students^16 20^ being the earliest stage at which participants were involved. In this sense, being able to compare the points of view of medical and dental students with those of CAs, clinicians and stakeholders involved in the management of training and careers is a point of novelty in our study. Our analysis confirms the finding, already presented in some of the studies,^2022 36^,^38^ that the difficulty of reconciling academic and clinical work is one of the main obstacles to CA barriers. As the other studies did not include medical or dental students, our analysis of the need for more information and opportunities for exposure to research opportunities and the CA career represents a novel contribution to the existing literature. Finally, some studies^16 20 22 35^ have reported on financial considerations that might make CA careers less attractive. However, as these studies focused from the PhD stage and up, the barriers have been mostly presented in terms of opportunity costs of delayed access to higher paid levels. Our study, including medical and dental students, shows that actual costs linked to training fees are also an important barrier for early exposure to research that is conducive to entering the CA pathway. These results build on previous studies on the costs of intercalating,^33 39^ which, however, were less linked to the broader issue of entry into the CA pathway.

### Future research

Further studies could explore such barriers with more significant numbers of participants, especially regarding medical and dental students and on a broader number of institutions. Survey studies involving a wider cohort of students could also build further knowledge about potential disparities in information about the CA pathway.

## Conclusion

Our findings show that better and more tailored information about academic career pathways should be provided to students. These findings also show that financial barriers can increase the difficulties in accessing CA pathways. Financial and systemic difficulties often intersect with social status and social class. This situation can discourage students from lower socioeconomic backgrounds from pursuing a CA career, adding to the barriers already identified in terms of class, gender and ethnicity.^27^ To better support medical students, it is essential to enhance the quality and quantity of information provided, especially in the early years, and address systemic obstacles related to the cost of additional training.

## Data Availability

No data are available.
