# Supplementary material for: Students’ and educators’ perceptions of clinical academic pathways in the UK: a qualitative study
Source: BMJ Open. 2025 Mar 24;15(3):e089791. doi: 10.1136/bmjopen-2024-089791 (PMC11934423; doi:10.1136/bmjopen-2024-089791)
Supplement: Supplementary file 1 [file bmjopen-2024-089791.reviewer_comments.pdf]

# PEER REVIEW HISTORY

BMJ Open publishes all reviews undertaken for accepted manuscripts. Reviewers are asked to complete a checklist review form and are provided with free text boxes to elaborate on their assessment. These free text comments are reproduced below.

## ARTICLE DETAILS

### Title (Provisional)

Students' and educators' perceptions of clinical academic pathways in the UK: a qualitative study

### Authors

Greco, Cinzia; Lin, Chiu-Yi; Gardiner, Natalie J; Finn, Gabrielle; Danquah, Adam; Radhakrishnan, Hema

---

## VERSION 1 - REVIEW

---

| | |
|---|---|
| **Reviewer** | **1** |
| **Name** | **Boushehri, Elham** |
| **Affiliation** | **Hormozgan University of Medical Sciences** |
| **Date** | **28-Jun-2024** |
| **COI** | **medical educationeducational assessment/planning** |

---

Thank you for the study you did. It is necessary to state that there are some fundamental flaws in your study, the most important of which I will mention as bellow:

In the second paragraph of the introduction section, I did not understand why the researcher was referring to these sentences: "To build long term trustful relationships with disadvantaged communities and reduce health inequalities through innovations in healthcare research it is important our CA cohort represents the diversity of the population it serves. It is evident that the women and individuals from ethnic minority backgrounds are still under-represented in the CA pathways". How do these two sentences relate to the rest of this paragraph? And what is its role in justifying the importance of this research?

In the last paragraph of the introduction section, I was expecting to read appropriate scientific or empirical reasons to justify the current study, but it was not available. The researcher referred to a study in the same area that had been done before, but according to his explanation, I did not see any difference between that study and the current study. Therefore, in my opinion, as a reader, referring to the previous study was not a convincing reason to conduct the current research.

In the Method and Introduction section, there are justifications that show why this study is not observed with a qualitative approach? Does the phenomenon under study require a deep and comprehensive study? Why?

The educational setting, the research community, the method of data collection and analysis, ethical considerations, research questions and the main questions of the interview sessions, the duration of interviews and trustworthiness not explained. In result section, the themes which were extracted, seem to be “category names” not themes.

---

| | |
|---|---|
| **Reviewer** | **2** |
| **Name** | **Trusson, Diane** |
| **Affiliation** | **University of Nottingham, School of Medicine** |
| **Date** | **09-Oct-2024** |
| **COI** | **There are no competing interests** |

---

This study explored students’ and educators’ perceptions of UK clinical academic pathways. Although it is an interesting read, I feel that the inclusion of just 6 medical/dental students who participated in two focus groups is too small for a study which is reporting students' perceptions. Also, I found the term ‘medical educators’ rather vague; it was not clear how many of the clinical academics, clinicians and stakeholders who were interviewed could be described as educators.

This research appears to be linked to a previous study conducted in the same location with what seems to be the same participants, but this is not explicitly stated.

I will now go through the manuscript with specific comments:

Page 2. Abstract. The conclusion needs to have more information (what were the conclusions?)

Line 35 There is a typo (strengths).

Line 45 This last point is a bit confusing, please reword it for clarification.

Page 3. The introduction seems very brief.

Line 14. The authors refer to objectives and principles developed by the UKCRC – what are they?

Line 15. If ‘significant attention has been paid to different clinical academic pathways’ then this suggests that there are other studies of enablers/barriers in clinical academia. These should be described here and referred to in the discussion.

Line 17. From medics to nurses does not seem very wide-ranging.

Line 36. Remove 'the' before 'women'.

Line 38. Underrepresented in the UK or generally? Only one of the references relates to the UK.

Line 50. Only one previous study of barriers and facilitators experienced by clinical academics has been cited and this seems to include the same sample as the current study. See previous comment for line 15. There should be greater discussion of what has been done before, and what gap in knowledge this study addresses.

Page 4. More details are needed about why qualitative methods were used.

Line 41. Who conducted the interviews/focus groups? What were the researchers' characteristics/personal attributes and any prior relationships with participants which may have affected the study?

Line 45. The dates of data collection should be reported in the results section.

Page 5. The description of the participants' characteristics does not include ethnicity even though it is highlighted as important in the introduction.

Line 36. How many students participated in each focus group?

Page 6. Line 10. The student says that they have 'not had information at all' even though the first line of the preceding paragraph states 'students […] were provided with information on CA careers.'

Lines 42-45. This is confusing for someone with little expertise in medical education and although it is explained in the discussion it might be worth also explaining before or after the quote.

Page 7. Line 34 is referring to costs which comes under theme 2.

Pages 10-11. The first paragraph of the discussion seems more relevant to the introduction section as it is not discussing any of the findings of the current study.

Page 11. Line 19 states that students receive information, but this is contrary to what the student interviewee says on page 6.

There is no discussion of theme 3.

Overall, the discussion needs to follow the guidance in the SRQR.

I hope that the above suggestions for improving the manuscript are helpful to the authors.

---

| | |
|---|---|
| **Reviewer** | **3** |
| **Name** | **Fawaz, Mirna** |
| **Affiliation** | **Beirut Arab University** |
| **Date** | **20-Dec-2024** |

**COI** **No competing interests.**

The paper is robust and can be accepted.

## VERSION 1 - AUTHOR RESPONSE

**Reviewer: 1**
**Dr. Elham Boushehri, Hormozgan University of Medical Sciences**

Q In the second paragraph of the introduction section, I did not understand why the researcher was referring to these sentences: “To build long term trustful relationships with disadvantaged communities and reduce health inequalities through innovations in healthcare research it is important our CA cohort represents the diversity of the population it serves. It is evident that the women and individuals from ethnic minority backgrounds are still under-represented in the CA pathways”. How do these two sentences relate to the rest of this paragraph? And what is its role in justifying the importance of this research?

A - We have added a passage to clarify that the aims that the CA community needs to pursue, including improving the relations with minoritised groups and reducing health inequalities, create a specific need not only to increase the number of CAs but also to diversify their composition.

Q- In the last paragraph of the introduction section, I was expecting to read appropriate scientific or empirical reasons to justify the current study, but it was not available. The researcher referred to a study in the same area that had been done before, but according to his explanation, I did not see any difference between that study and the current study. Therefore, in my opinion, as a reader, referring to the previous study was not a convincing reason to conduct the current research.

A- We have not clarified further that, while the first publication explored general barriers and facilitators in the careers of CAs, the present analysis looks in particular at the role of medical education, which we have not covered in the first publication. We have also expanded across the Introduction on the relevance of our analysis.

Q- In the Method and Introduction section, there are justifications that show why this study is not observed with a qualitative approach? Does the phenomenon under study require a deep and comprehensive study? Why?

A -As mentioned above we have expanded further on the importance of studying factors facilitating the recruitment of CAs and their careers; our study is indeed qualitative, as we have clarified in both version of the article.

**Reviewer: 2**

**Dr. Diane Trusson, University of Nottingham**

Q I feel that the inclusion of just 6 medical/dental students who participated in two focus groups is too small for a study which is reporting students' perceptions.

A We recognise that the sample of medical and dental students is limited, but we think that it is important to compare data deriving from both students and stakeholders, given the important role we identify for medical education

Q Also, I found the term 'medical educators' rather vague; it was not clear how many of the clinical academics, clinicians and stakeholders who were interviewed could be described as educators.

A - All the participants classified as medical educators had a role of medical education – some have a range of managerial responsibilities linked to the organisation of medical education, and most are directly involved in teaching medical and dental students. We have opted not to expand on this aspect in order to avoid making individual participants identifiable.

Q- This research appears to be linked to a previous study conducted in the same location with what seems to be the same participants, but this is not explicitly stated.

A- We have now added a passage towards the end of the Introduction confirming this point more explicitly

Q Page 2. Abstract. The conclusion needs to have more information (what were the conclusions?)

A We have made this passage more explicit

Q Page 3. The introduction seems very brief.

A We have now extended the Introduction, addressing the suggestions of the review

Q Line 14. The authors refer to objectives and principles developed by the UKCRC – what are they?

A We have now added this detail

Q Line 50. Only one previous study of barriers and facilitators experienced by clinical academics has been cited and this seems to include the same sample as the current study. See previous comment for line 15. There should be greater discussion of what has been done before, and what gap in knowledge this study addresses.

A We have added a specific section to discuss existing literature and our contribution in relation to this literature, in the Discussion

Q Page 4. More details are needed about why qualitative methods were used.

A We have now clarified that the exploratory nature of the study and the relatively limited size of the population of reference motivated the choice of a qualitative approach

Q Line 41. Who conducted the interviews/focus groups? What were the researchers' characteristics/personal attributes and any prior relationships with participants which may have affected the study?

A We had indicated that the first two authors have conducted the interviews and focus groups, and we have now further clarified that the researchers are not involved in medical training and had no prior relationship with the participants.

Q Page 5. The description of the participants' characteristics does not include ethnicity even though it is highlighted as important in the introduction.

A We have now added this aspect

Q Page 6. Line 10. The student says that they have 'not had information at all' even though the first line of the preceding paragraph states 'students [...] were provided with information on CA careers.'

A From a closer examination of the focus group transcript (which however is difficult to reproduce in the space of the article) we can confirm that this passage was hyperbolic and that the same participant elsewhere discusses receiving the information in question, even in less detail than they would have liked to have

Q Lines 42-45. This is confusing for someone with little expertise in medical education and although it is explained in the discussion it might be worth also explaining before or after the quote.

A We have now added a short explanation of the passage immediately after the quote

Q Page 7. Line 34 is referring to costs which comes under theme 2.

A We agree that this passage touches upon another theme, but we have opted to keep the unity of the participant's discourse even if it transgresses in part the division in themes

Q Pages 10-11. The first paragraph of the discussion seems more relevant to the introduction section as it is not discussing any of the findings of the current study.

A We have moved the passage in question in the Introduction

Q Overall, the discussion needs to follow the guidance in the SRQR.

A We have expanded significantly the discussion, making sure to incorporate the different points of the SRQR

---

## VERSION 2 - REVIEW

---

| | |
|---|---|
| **Reviewer** | **2** |
| **Name** | **Trusson, Diane** |
| **Affiliation** | **University of Nottingham, School of Medicine** |
| **Date** | **15-Feb-2025** |
| **COI** | |

---

Thank you for addressing all the comments and recommendations from the previous review.

There is one very minor amendment that I would request. The conclusion part of the abstract would be clearer if it was two sentences. And rather than saying 'reduces the attractiveness' it might be better to reword it along the lines of ' The overall cost of training for students makes the clinical academic pathway less attractive/less appealing'.

Otherwise I'm happy to recommend that the manuscript is accepted for publication.
